# Kawasaki disease presenting with severe airway obstruction: a case report

**DOI:** 10.3389/fped.2026.1797418

**Published:** 2026-04-14

**Authors:** Yunfeng Xiang, Daoxue Xiong

**Affiliations:** Department of Pediatric Critical Care, Chongqing University Three Gorges Hospital, Chongqing, Wanzhou, China

**Keywords:** Kawasaki disease, airway obstruction, epiglottitis, respiratory distress, a case report

## Abstract

**Background:**

Kawasaki disease is an acute systemic vasculitis primarily affecting children, typically presenting with persistent fever, rash, conjunctival injection, and oral mucosal changes. Although pharyngeal involvement may occur, severe upper airway obstruction requiring mechanical ventilation is extremely rare. We report a pediatric case of Kawasaki disease manifesting primarily as epiglottitis and laryngeal obstruction.

**Case presentation:**

A 15-month-old boy presented with a 6-day history of persistent fever and a 1-day history of neck swelling and progressive dyspnea. At the time of admission, the child showed signs of laryngeal obstruction. Other findings were a rash, enlarged cervical lymph nodes, mild redness of the conjunctiva, cracked lips, and a strawberry tongue. Neck CT demonstrated extensive wall thickening and edema in the nasopharynx, oropharynx, and larynx. Fiberoptic laryngoscopy revealed extensive white pseudomembranes on the posterior pharyngeal wall and a congested, swollen, spherical epiglottis. During hospitalization, progressive respiratory distress required endotracheal intubation and mechanical ventilation. Sputum culture identified *methicillin-resistant Staphylococcus aureus*. The patient's condition stabilized after combination therapy with intravenous immunoglobulin, vancomycin, and aspirin. In the convalescent phase, thrombocytosis and desquamation of the fingertips and toes were observed, fulfilling the clinical diagnostic criteria for Kawasaki disease.

**Conclusion:**

Although Kawasaki disease rarely causes epiglottitis, it should be considered as a differential diagnosis in children presenting with fever, neck swelling, and laryngeal obstruction. Early recognition and prompt initiation of immunomodulatory therapy can effectively control disease progression and prevent unnecessary surgical intervention.

## Introduction

Kawasaki disease is an acute febrile illness primarily affecting children under 5 years of age. It has become the leading cause of acquired heart disease in children in developed countries (1). The typical clinical features of Kawasaki disease include persistent fever, rash, cervical lymphadenopathy, non-exudative conjunctivitis, lip fissures, and erythema with edema of the hands and feet. A minority of children may also present with arthritis, aseptic meningitis, pyuria, or gastrointestinal symptoms (2). In recent years, cases of Kawasaki disease presenting with parapharyngeal or retropharyngeal space cellulitis or abscess have been increasingly reported, However, progression to severe airway obstruction remains uncommon (3, 4). We present the case of a 15-month-old child who, during the course of Kawasaki disease, developed extensive peripharyngeal tissue swelling accompanied by abundant white pseudomembranes on the posterior pharyngeal wall and severe epiglottic swelling, ultimately requiring mechanical ventilation for progressive laryngeal obstruction. Cases of severe epiglottic and laryngeal edema directly attributable to Kawasaki disease requiring mechanical ventilation are extremely rare in the literature.

## Case presentation

A 15-month-old boy was admitted with a 6-day history of fever and a 1-day history of bilateral neck swelling, dysphagia, and dyspnea. Two days before admission, he received intravenous piperacillin-tazobactam at a local county hospital; however, the fever persisted.

On admission, the patient's vital signs were as follows: temperature 39.4 °C, heart rate 164 bpm, respiratory rate 42 breaths/min, blood pressure 98/54 mmHg, and oxygen saturation 90% on room air. He was a 9.6 kg male infant. He was alert but appeared lethargic and was intermittently irritable. He had dyspnea with open-mouth breathing. Audible snoring, inspiratory stridor, and marked retractions in the suprasternal and subcostal areas were present. Perioral cyanosis was noted. A scattered dark-red rash was seen on his face and trunk. The neck was diffusely swollen bilaterally with multiple enlarged lymph nodes palpable. Mild bilateral periorbital edema and conjunctival injection were present. The lips were dry with fissures, and a strawberry tongue was noted. Oropharyngeal examination revealed diffuse erythema with whitish pseudomembranous patches on the posterior pharyngeal wall. Lung auscultation demonstrated coarse breath sounds bilaterally without crackles or wheezes. Cardiac and abdominal examinations were unremarkable. There was no erythema or edema of the fingers or toes.

**Figure 1:**
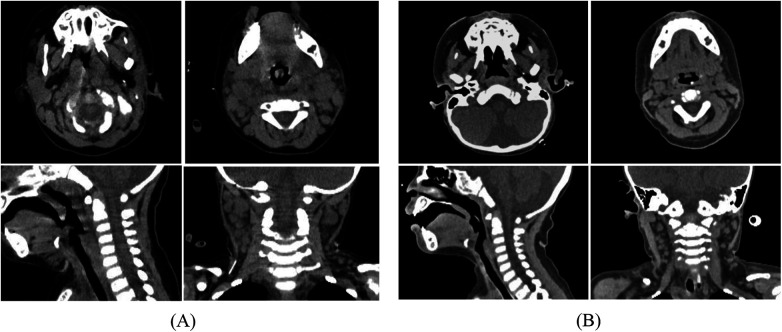
**(A)**: Neck CT at admission shows extensive swelling and thickening of the nasopharyngeal, oropharyngeal, and laryngeal walls with narrowing of the laryngeal cavity; multiple enlarged lymph nodes in bilateral neck regions II, III, and IB with blurred surrounding fat planes. **(B)**: Follow-up cervical CT scan on hospital day 7 showing marked reduction in the size of cervical and submandibular lymph nodes and resolution of parapharyngeal and retropharyngeal swelling.

**Figure 2:**
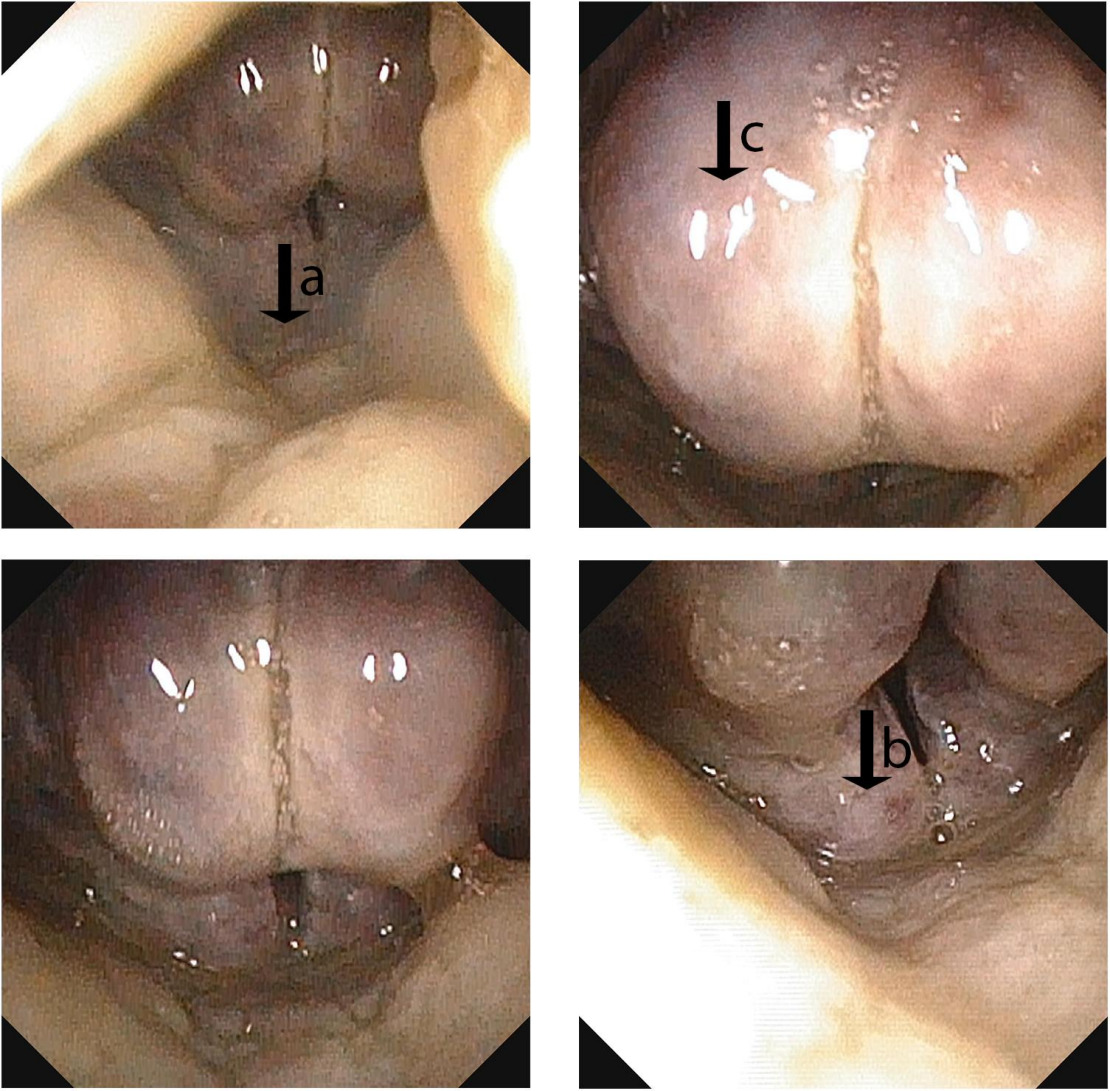
Flexible laryngoscopy on admission showing hyperemic and swollen mucosa of the posterior pharyngeal wall and larynx(↓a), covered with extensive white pseudomembranes(↓b). The epiglottis is hyperemic, swollen, and spherical(↓c).

Laboratory and imaging findings on admission were as follows: Arterial blood gas analysis revealed pH 7.38, pCO₂ 27.5 mmHg, pO₂ 69.0 mmHg, oxygen saturation 95%, lactate 3.6 mmol/L, and base excess −7.6 mmol/L. Complete blood count showed leukocytosis (10.62 × 10⁹/L) with neutrophilia (82%), lymphopenia (10%), and 4% atypical lymphocytes; hemoglobin was 107 g/L and platelet count 162 × 10⁹/L. Inflammatory markers were significantly elevated: C-reactive protein 113.9 mg/L and procalcitonin 4.73 ng/mL. Cervical CT demonstrated extensive swelling and thickening of the nasopharyngeal, oropharyngeal, and laryngeal walls, resulting in a narrowed laryngeal lumen. There was thickening of the adjacent fascial planes with a small amount of fluid in the fascial spaces. Multiple enlarged lymph nodes with blurred surrounding fat planes were noted in the bilateral cervical and submandibular regions (Figure 1A). Flexible laryngoscopy revealed congested and edematous mucosa of the posterior pharyngeal wall and larynx, covered with extensive white pseudomembranes. The epiglottis was congested and swollen, assuming a spherical shape. The vocal cords and subglottic area could not be visualized due to the severity of edema and exudate (Figure 2). Echocardiogram: No significant abnormalities noted in cardiac morphology and structure. Left main coronary artery (LMCA) diameter: 2.0 mm (Z-score 0.66); Left anterior descending artery (LAD) diameter: 1.5 mm (Z-score 0.06); Right coronary artery (RCA) diameter: 1.7 mm (Z-score 0.57).

Upon admission, the differential diagnoses included Kawasaki disease, cervical cellulitis, and infectious mononucleosis. Initial Treatment: Heated humidified high-flow nasal cannula oxygen therapy; Vancomycin (0.15 g IV q8h); Methylprednisolone (20 mg IV q12h). However, the child's laryngeal obstruction progressed with marked respiratory distress. Oxygen saturation gradually decreased to 80%–90%, accompanied by persistent tachycardia of 180–200 beats per minute. Consequently, on the day of admission, he underwent orotracheal intubation and mechanical ventilation (a 3.5-mm endotracheal tube was selected due to significant epiglottic swelling). Intravenous immunoglobulin (2 g/kg) was administered, and aspirin (30 mg/kg per day) was added.

**Figure 3:**
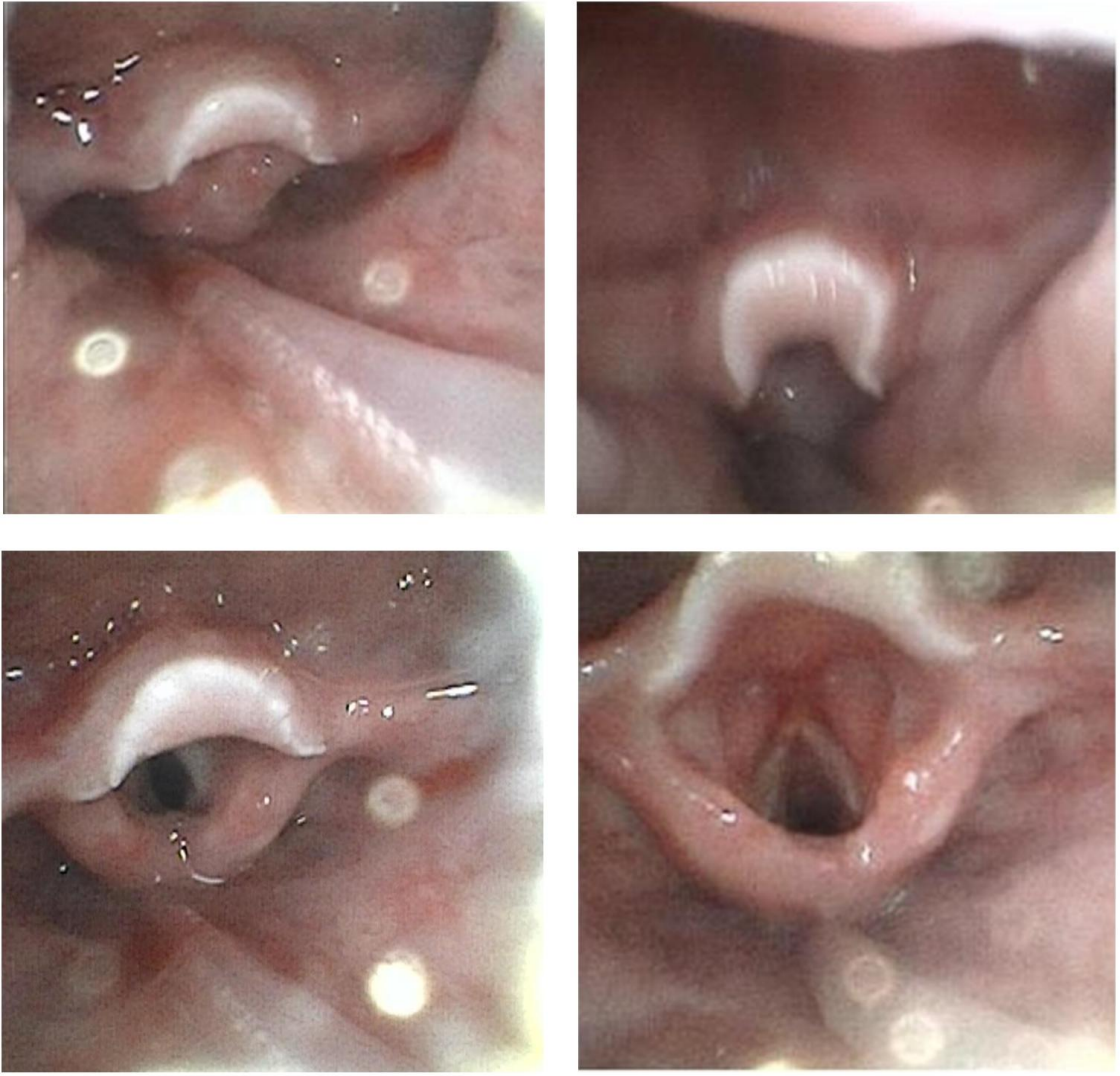
Fiberoptic bronchoscopy on hospital day 9 showing significant resolution of swelling in the posterior pharyngeal wall, epiglottis, and larynx, with disappearance of the white pseudomembranes.

On the next day of admission, the patient's body temperature normalized and neck swelling decreased. Laboratory tests showed essentially normal liver and renal function, as well as a normal coagulation profile. Tests for Epstein–Barr virus antibodies and DNA were negative. On hospital day 4, the patient was successfully weaned from mechanical ventilation. The aspirin dose was reduced to 3 mg/kg per day, and intravenous methylprednisolone was switched to oral prednisone at 2 mg/kg per day. A sputum culture from hospital day 3 grew methicillin-resistant Staphylococcus aureus. Intravenous vancomycin was continued for a total of one week. A follow-up CT scan on hospital day 7 showed significant reduction in the size of cervical and submandibular lymph nodes and marked improvement in parapharyngeal and retropharyngeal swelling (Figure 1B). Fiberoptic bronchoscopy on hospital day 9 showed substantial resolution of swelling in the posterior pharyngeal wall, epiglottis, and larynx, with complete disappearance of the white pseudomembranes (Figure 3). During hospitalization, thrombocytosis developed, with a peak platelet count of 1,256 × 10⁹/L, which later gradually decreased. Desquamation of the fingers and toes appeared in the second week, confirming the clinical diagnosis of Kawasaki disease. The prednisone dose was tapered and discontinued over 15 days. Echocardiography during hospitalization was normal. At one-month follow-up after discharge, echocardiography revealed a left main coronary artery (LMCA) diameter of 2.0 mm (Z-score 0.52), a left anterior descending artery (LAD) diameter of 1.2 mm (Z-score −1.29), and a right coronary artery (RCA) diameter of 1.6 mm (Z-score 0.09). The patient continues to take oral aspirin and is under regular outpatient follow-up.

## Discussion

Kawasaki disease is an acute systemic vasculitis that predominantly affects children. Without timely diagnosis and treatment, it can lead to coronary artery lesions. The early administration of intravenous immunoglobulin combined with aspirin has been shown to significantly reduce the risk of developing coronary artery lesions (5). According to the latest guidelines from the American Heart Association (AHA), the diagnostic criteria for Kawasaki disease include fever persisting for at least 4 days, along with at least four of the following five principal clinical features:(1) polymorphic rash; (2) bilateral non-exudative conjunctival injection; (3) oral changes: erythema and cracking of the lips, strawberry tongue, or oropharyngeal mucosal injection; OR Changes in the extremities: erythema of the palms and soles (usually accompanied by swelling) with periungual desquamation in the subacute phase;(4) cervical lymphadenopathy (typically unilateral, with a diameter ≥1.5 cm); (5) exclusion of other known diseases that may explain the presentation (1).

This patient presented with fever that did not respond to outpatient intravenous antibiotics. Upon admission, he had been febrile for 6 days and presented with rash, cervical lymphadenopathy, conjunctival injection, and lip fissures. These findings were consistent with the clinical features of Kawasaki disease. However, the conjunctival injection and lip fissures in this patient were mild. The presence of significant upper airway obstruction along with extensive pseudomembranes on the posterior pharyngeal wall complicated the initial differential diagnosis, raising the possibility of infectious etiologies such as cervical cellulitis or infectious mononucleosis. To clarify the cause, *Epstein–Barr virus (EBV)* serology was performed and returned negative, thereby ruling out infectious mononucleosis. On day 3 of hospitalization, sputum culture grew *methicillin-resistant Staphylococcus aureus*. It has been proposed that the pathogenesis of Kawasaki disease may be associated with immune activation triggered by superantigens from certain microorganisms, such as Staphylococcus aureus (6). In this case, the patient did not respond well to initial piperacillin-tazobactam treatment. However, after receiving intravenous immunoglobulin, his fever rapidly resolved and his neck swelling significantly subsided. This clinical course suggested that bacterial infection was not the primary etiology, and that *methicillin-resistant Staphylococcus aureus* was more likely an incidental colonizer. In the later stages of the disease course, the patient exhibited marked thrombocytosis and desquamation of the fingertips and toes, clinically consistent with the typical progression of Kawasaki disease, ultimately confirming the diagnosis. Nevertheless, given the patient's severe airway obstruction, epiglottic swelling, and intense systemic inflammatory response, we used intravenous immunoglobulin and aspirin as the foundation of treatment. In addition, we administered a full one-week course of vancomycin for anti-infective therapy and short-term methylprednisolone to control laryngeal edema and systemic inflammation. This approach played a key role in controlling disease progression.

Cervical involvement in Kawasaki disease typically presents as lymphadenopathy, usually unilateral and predominantly confined to the anterior cervical triangle. Since Bradley et al. (7) first reported Kawasaki disease with retropharyngeal masses in 1983, multiple studies have confirmed that this condition can involve deep cervical spaces, causing parapharyngeal or retropharyngeal edema and even abscess formation. The mechanism may be related to systemic vasculitis and increased microvascular permeability, leading to tissue fluid leakage and inflammatory cell infiltration. Clinically, such lesions often mimic deep neck infections, frequently leading to misdiagnosis (8). However, it is important to note that Kawasaki disease causes a cellulitis-like edema, not a true bacterial abscess. The treatment for these two conditions is very different. Surgical intervention should be considered with caution in Kawasaki disease. Therefore, Kawasaki disease should be considered in children who have deep neck lesions, persistent high fever, and do not respond to antibiotic therapy (9). A contrast-enhanced CT scan is useful to help tell the difference. It can distinguish Kawasaki disease-related cervical lymphadenopathy and retropharyngeal edema from suppurative lymphadenitis and retropharyngeal abscess (10). Due to the urgent clinical condition and incomplete renal function assessment at admission, a non-contrast CT was performed in this patient. Although it clearly showed the extent of soft tissue swelling, it had certain limitations in differential diagnosis.

According to the literature, deep neck space edema caused by Kawasaki disease can sometimes compress the airway (11). However, it is very rare for this to lead to severe airway obstruction requiring mechanical ventilation or even a tracheostomy. A previous report by Burgner et al. described a 4-year-old child with Kawasaki disease who had severe dysphagia and respiratory distress requiring endotracheal intubation (12). In our case, the child developed clear signs of laryngeal obstruction during the illness. A fiberoptic laryngoscopy was performed. It showed that the epiglottis was severely congested and swollen, appearing spherical. There were also extensive pseudomembranes covering the posterior pharyngeal wall. These findings resulted in severe narrowing of the laryngeal airway. The condition progressed to respiratory failure. Therefore, emergency endotracheal intubation and mechanical ventilation were necessary. For managing the airway, we noted significant swelling in the area above the vocal cords and a narrowed laryngeal opening. For this reason, we selected an endotracheal tube with a smaller internal diameter (3.5 mm) than typically used for a child of this age. This choice was made to reduce the risk of injury during intubation and to ensure effective ventilation. A review of the literature shows that reports of Kawasaki disease directly causing severe epiglottic and laryngeal edema requiring artificial airway establishment are extremely limited. This case provides an important reference for clinical practice.

## Summary

This case highlights that clinicians should remain highly vigilant and promptly consider Kawasaki disease in the differential diagnosis for febrile children presenting primarily with neck swelling and laryngeal obstruction, even in the absence of typical clinical symptoms. Integrating imaging studies with laryngoscopy facilitates early identification of deep cervical space edema and epiglottitis, thereby preventing misdiagnosis as a simple cervical infection. Timely administration of intravenous immunoglobulin and anti-inflammatory therapy can significantly improve airway edema and control systemic inflammation. This is crucial for preventing coronary artery complications and alleviating airway crises. For children presenting with severe laryngeal obstruction, prompt airway intervention such as endotracheal intubation is essential for saving lives.

## Data Availability

The original contributions presented in the study are included in the article/Supplementary Material, further inquiries can be directed to the corresponding author.
